# Long-Term Peritoneal Dialysis Using a Tenckhoff Catheter in a Premature Infant With Homozygous Autosomal Recessive Polycystic Kidney Disease: A Case Report

**DOI:** 10.7759/cureus.87958

**Published:** 2025-07-14

**Authors:** Maria Florou, Kleanthis Anastasiadis, Maria Tsopozidi, Chrysostomos Kepertis, Vassileios Lambropoulos, Vassileios Mouravas, Christos Kaselas

**Affiliations:** 1 2nd Department of Pediatric Surgery, Aristotle University of Thessaloniki, Papageorgiou General Hospital, Thessaloniki, GRC; 2 Department of Pediatric Surgery, Ippokrateio General Hospital, Thessaloniki, GRC; 3 Department of Pediatric Surgery, Larissa General University Hospital, Larissa, GRC; 4 1st Department of Pediatric Surgery, Aristotle University of Thessaloniki, Gennimatas General Hospital, Thessaloniki, GRC

**Keywords:** infant, peritoneal dialysis, polycystic kidney disease, renal failure, tenkchoff catheter

## Abstract

Polycystic kidney disease (PKD) is a clinical condition characterized by the presence of renal cysts and kidney enlargement. When it presents during the neonatal period, it can lead to progressive renal damage and eventually result in end-stage renal failure. Peritoneal dialysis (PD) can be particularly challenging in the neonatal intensive care unit due to the infant's thin abdominal wall and the often unsuitable size and shape of available catheters. As a result, the use of such catheters in infants is associated with high mortality and significant complications, including leakage, peritonitis, and hemoperitoneum. Here, we report our initial experience with Tenckhoff catheter placement and its management over 72 days in a premature newborn diagnosed with autosomal recessive polycystic kidney disease. To the best of our knowledge, this is among the few reported cases where PD was successfully maintained with a Tenckhoff catheter for such an extended duration. Despite the challenges posed by low body weight and a thin abdominal wall, PD remains a relatively effective and feasible treatment option for neonates with renal failure.

## Introduction

Polycystic kidney disease (PKD) is characterized by multiple renal cysts originating from the proximal tubules. These cysts are benign lesions filled with serous fluid and remain connected to the nephrons [1]. Based on inheritance, PKD is classified as either autosomal dominant PKD (ADPKD) or autosomal recessive PKD (ARPKD) [2]. In infants, PKD can lead to progressive renal damage and may result in end-stage renal failure, requiring renal replacement therapy during infancy or early childhood [3,4]. The main renal replacement therapies in neonates include intermittent hemodialysis, peritoneal dialysis (PD), and continuous renal replacement therapy. Among these, continuous PD is currently considered the most feasible method for newborns. However, placing a dialysis catheter is technically challenging due to the mismatch between catheter size and the infant's thin abdominal wall. This is even more difficult in premature infants, who have lower body weight, scant subcutaneous fat, a very thin abdominal wall, and less flexible skin. Additionally, standard Tenckhoff catheters may be poorly suited in size and shape for this population. As a result, catheter placement in premature infants is technically demanding and carries a high complication rate, ranging from 20% to 60%, with catheter malfunction, leakage, and peritonitis being the most common issues [5].

Here, we report our experience with Tenckhoff catheter placement and 72-day management in a premature infant with renal failure secondary to autosomal recessive PKD. To the best of our knowledge, this is one of the few reported cases in which PD was successfully maintained using a Tenckhoff catheter over such a long period. As there are limited reports describing this procedure in neonates with ARPKD, we believe this case contributes valuable insight to the existing literature.

## Case presentation

A female newborn with a gestational age of 35 + 4/7 weeks and a birth weight of 2415 g was admitted to the neonatal intensive care unit (NICU) immediately after birth due to prematurity, respiratory distress syndrome, and PKD. The Apgar scores were 7 at both the 1st and 5th minutes. The infant was intubated in the delivery room, and conventional mechanical ventilation was initiated. PKD had first been detected on a second-level ultrasound at 25 weeks of gestation, along with oligohydramnios that progressed to anhydramnios by the time of delivery. Amniocentesis performed at 30 weeks revealed homozygosity for a mutation in the polycystic kidney and hepatic disease-1 (PKHD1) gene, confirming ARPKD. At birth, the newborn displayed phenotypic features consistent with Potter-like syndrome, including a flattened nose, small dysplastic low-set ears, bowed legs, and clubbed feet. Abdominal ultrasound confirmed the presence of polycystic kidneys, without evidence of hepatic involvement. The neonate was oliguric from the first day of life. Furosemide was administered, but renal function rapidly deteriorated.

**Figure 1:**
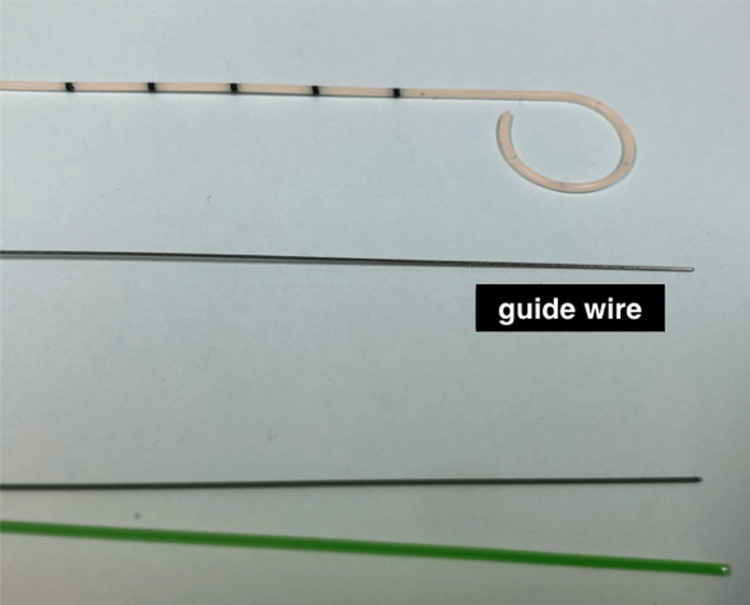
A guide wire from a pig-tail catheter was inserted into the Tenckhoff catheter tube to assist with its repositioning in the NICU.

Due to worsening respiratory and metabolic acidosis, electrolyte imbalance, and generalized edema, PD was indicated. A 31 Fr neonatal straight single-cuff Tenckhoff catheter (Cook Medical, Bloomington, USA), 8.5 cm in length, was placed bedside in the NICU by a pediatric surgeon from our department, as the infant was too unstable for transfer to the operating room. A right paramedian incision was made near the umbilicus, and the subcutaneous tissues were dissected down to the rectus sheath. The catheter was inserted through the peritoneum and directed toward the pelvis. Dialysis was initiated on day 15, with daily peritoneal lavages containing heparin, vancomycin, and ceftazidime. On the 8th day of lavage, catheter displacement from the Douglas pouch was detected. The catheter was repositioned in the operating room, and partial omentectomy was performed due to omental obstruction of the catheter’s distal tip. After repositioning, the infant was gently rotated daily to the right or left to facilitate drainage and prevent further occlusions. On several occasions, the catheter’s distal end required adjustment at bedside by the pediatric surgeon, using the guide wire of a pig-tail catheter to improve dialysis function (Figure 1).

**Figure 2:**
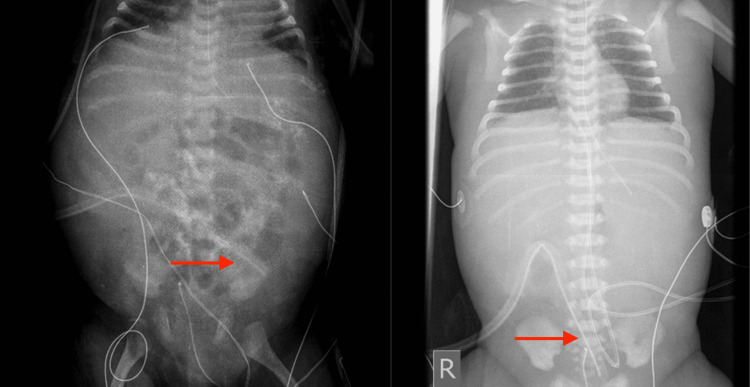
Abdominal X-ray on the 16th and the 73rd day of life showing the positioned Tenckhoff catheter. Red arrows indicate the catheter.

In total, the newborn was hospitalized in the neonatal unit for 82 days, with peritoneal lavages performed there for 67 days. On the 82nd day of life, the infant was transferred to the pediatric clinic for nephrology management, where peritoneal lavages continued for an additional 15 days. Gentle side-to-side positioning of the infant was maintained during dialysis to support catheter function. No leakage of peritoneal fluid from the catheter exit site or signs of infection were observed throughout the procedure. The infant was closely monitored through regular clinical assessments and laboratory tests, including inflammatory markers and cultures of blood and peritoneal fluid. Fortunately, no signs of inflammation or infection were detected (Figure 2).

The patient was never discharged from the pediatric clinic, as she passed away one month later due to a severe nosocomial respiratory infection that progressed to sepsis. However, she survived renal failure, two surgical procedures for Tenckhoff catheter placement, and prolonged PD, without the morbidity typically reported in the literature.

## Discussion

PKD is characterized by multiple renal cysts originating from the proximal tubules, along with kidney enlargement. It can present in utero, during infancy, or in childhood, and may affect one or both kidneys. Based on inheritance patterns, PKD is classified as either autosomal dominant (ADPKD) or autosomal recessive (ARPKD) [2]. The autosomal recessive form is rare, with an incidence of approximately 1 in 20,000 live births. It is typically diagnosed prenatally or shortly after birth and may lead to progressive renal deterioration and eventual end-stage renal failure, requiring renal replacement therapy and, ultimately, kidney transplantation [3,4]. PD is considered the most suitable form of renal replacement therapy, even for the smallest infants. However, several technical challenges must be overcome when placing the dialysis catheter in neonates. These include low body weight, scant subcutaneous fat, a thin abdominal wall, inelastic skin, and the incompatibility between standard Tenckhoff catheter dimensions and the anatomy of infants. Consequently, PD in this population is associated with a high complication rate, ranging from 20% to 60%, with greater prematurity and lower birth weight linked to increased risk [5]. Recently, two large retrospective studies by Maizlin et al. and Hakan et al. evaluated PD complications in neonates. The most commonly reported issues included catheter leakage, malfunction or obstruction, peritonitis, wound infection, and, less frequently, bleeding during insertion or bowel perforation [6,7]. A 2023 case report by Jiang et al. also described intestinal prolapse following Tenckhoff catheter placement in a low-birth-weight infant [8]. Significant mortality rates have been noted in neonates undergoing PD, though often in the presence of comorbidities or other contributing factors [6,7]. In our case, Tenckhoff catheter obstruction was resolved through surgical repositioning and partial omentectomy. Potential subsequent malfunctions were likely prevented by daily gentle repositioning of the infant to the right or left side before each dialysis session, as well as by bedside catheter adjustments using a guide wire performed several times by the pediatric surgeon. These measures appeared to facilitate the dialysis process, which was successfully continued for 72 days. To our knowledge, this represents the longest reported duration of PD using a Tenckhoff catheter in a premature infant. A recent literature review [9] identified the longest previously reported PD duration as 70 days, achieved with a thoracic drain tube rather than a Tenckhoff catheter [10]. Despite requiring an omentectomy, the prolonged and effective dialysis achieved in our case may be attributed to careful daily patient positioning and the timely bedside catheter repositioning with the guide wire by the pediatric surgical team.

## Conclusions

In conclusion, neonatal PKD is associated with significant morbidity and mortality. Despite technical challenges related to low birth weight, a thin abdominal wall, and minimal subcutaneous fat, PD remains a practical and effective treatment option for neonates with renal failure, even in the absence of a catheter specifically sized for this population. Simple interventions, such as daily repositioning of the infant before each lavage and catheter adjustments using guide wires, appear to support the long-term success of the procedure. Close collaboration between pediatric surgeons and pediatricians is essential for both the placement and ongoing management of the Tenckhoff catheter to optimize outcomes and minimize complications.
